# DF-dRVFL: A novel deep feature based classifier for breast mass classification

**DOI:** 10.1007/s11042-023-15864-2

**Published:** 2023-07-11

**Authors:** Xiang Yu, Zeyu Ren, David S. Guttery, Yu-Dong Zhang

**Affiliations:** 1 School of Computing and Mathematical Sciences, University of Leicester, University Road, Leicester, LE1 7RH Leicestershire UK; 2 Leicester Cancer Research Centre, University of Leicester, University Road, Leicester, LE2 7LX Leicestershire UK

**Keywords:** Breast mass classification, Deep learning, Transfer learning, ELM, RVFLN, SNN

## Abstract

Amongst all types of cancer, breast cancer has become one of the most common cancers in the UK threatening millions of people’s health. Early detection of breast cancer plays a key role in timely treatment for morbidity reduction. Compared to biopsy, which takes tissues from the lesion for further analysis, image-based methods are less time-consuming and pain-free though they are hampered by lower accuracy due to high false positivity rates. Nevertheless, mammography has become a standard screening method due to its high efficiency and low cost with promising performance. Breast mass, as the most palpable symptom of breast cancer, has received wide attention from the community. As a result, the past decades have witnessed the speeding development of computer-aided systems that are aimed at providing radiologists with useful tools for breast mass analysis based on mammograms. However, the main issues of these systems include low accuracy and require enough computational power on a large scale of datasets. To solve these issues, we developed a novel breast mass classification system called DF-dRVFL. On the public dataset DDSM with more than 3500 images, our best model based on deep random vector functional link network showed promising results through five-cross validation with an averaged AUC of 0.93 and an average accuracy of $81.71\%$. Compared to sole deep learning based methods, average accuracy has increased by 0.38. Compared with the state-of-the-art methods, our method showed better performance considering the number of images for evaluation and the overall accuracy.

## Introduction

Breast cancer is the most frequent cancer among women in the UK. While most breast cancer diagnoses occur in women over 50, and younger women can also develop breast cancer. About 1 in 8 women are diagnosed with breast cancer during their lifetime, but there is a good chance of recovery if detected early [34]. Therefore, there are lots of researchers who focus on how to early detect breast cancer in the early stage by computer-aided (CAD) systems. CAD systems can be subdivided into computer-aided detection (CADe) systems and computer-aided diagnosis (CADx) systems. The CADe systems are mainly utilized for extracting ROIs from medical images for further analysis tasks. Based on the obtained ROIs, the CADx systems focus on extracting features from the obtained ROIs and make predictions of severity based on the extracted features. There are several challenges to the early detection of breast cancer by CAD systems. Firstly, compared to natural images, mammograms are usually with higher resolutions and larger sizes. The high resolutions are challenging for both the hardware and the performance of the algorithms for diagnosis. Secondly, the anatomical architectures of the organs and the tissues in mammograms are more difficult to recognize and detect than the natural images. Traditional CAD systems cannot easily obtain these features of anatomical architectures.

In recent years, with the development of deep learning, deep learning-based CAD systems achieved great results in solving existing challenges of CAD systems. However, deep learning-based CAD systems also have some limitations for breast mass classification tasks. Firstly, the performance of the deep learning model highly relies on differentiating malignant masses from benign masses. The main differences between benign and malignant masses are the shape and margins of the masses [1]. The shape of a mass can likely be irregular, round, and lobular, while benign masses are more likely to have circumscribed oval and round shapes, and malignant masses tend to have irregular shapes. The margins of the masses can also be subdivided into categories, including microlobulated, obscured, ill-defined, and spiculated. The microlobulated margins describe the scalloped appearance of the mass that is distinct from the breast tissues. The obscured margins indicate the margins of the masses that were partially blocked by adjacent tissue. As a result, the masses in this situation may fail to be differentiated from the breast tissue. The ill-defined margins refer to the margins that are indistinct from the breast tissue. The reason why margins are ill-defined can be the low contrast of the images and the high breast density. The spiculated margins are shown in the form of radiating lines from the breast masses and are usually shown in malignant breast masses. However, malignant masses can also have circumscribed margins with a low possibility that follow-up examinations are required to distinguish those masses. The second limitation is that deep learning models always need sufficient computational power, and the improvements of well-trained models are hard to obtain, although there are powerful computational resources.

Based on the challenges and limitations mentioned above, it is still of great value to design a deep learning-based CADx system for breast mass classification as they can advise radiologists in a relatively short time and facilitate the diagnosis procedures instead of forcing patients to go through painful tissue extraction procedures for biopsy. Aimed at developing a breast mass classification system with promising performance, we proposed to develop a novel deep feature-based model called DF-dRVFL. The main contributions of this study can be concluded as follows:

1. We developed a high-performance CADx system for breast mass classification based on DF-dRVFL for mammography images. The developed system works on the extracted ROIs using a previously developed breast mass detection system. The developed system consists of three components: model training, feature extraction, and feature classification. In model training, we transferred the state-of-the-art deep models that were pre-trained on ImageNet instead of training the models from scratch. We first removed the top layers of the deep models as they were initially trained for 1,000-class classification and added new fully connected layers for the classification task here. We also added the dropout layers to prevent the trained models from overfitting. After training, we then extracted the features from the trained models as the input of our classifier DF-dRVFL. The experimental results on the public DDSM dataset showed the effectiveness of the developed system and therefore validated the plausibility between the combinations of breast mass feature extraction and classification.
2. We developed a novel strategy for breast mass classification by introducing a novel hybrid deep learning-based model. In this study, we proposed a VGG19-DF to deploy trained deep learning models as feature extractor instead of relying on hand-crafted features. As mentioned before, the differences between benign masses and malignant masses mainly lie in the shapes and the margins. However, these hand-crafted features are not reliable, and they can mislead the diagnosis results. Instead, we proposed to use deep features that are extracted by a trained deep learning model for mass classification. Compared to hand-crafted features, deep features are more representative and robust, and the models trained with deep features are likely to show higher performance.
3. We found an efficient method for breast mass classification performance improvement with low computational cost. If well fine-tuned, the performance of deep learning models can be improved step by step. However, the process pf optimizing the settings of hyper-parameters for model optimization can be lengthy. Therefore, there is an unmet need to improve the performance of the classifiers at a minimal cost. Toward this, we proposed to introduce DF-dRVFL for fast performance improvement. Experiments on the public dataset DDSM showed that the novel classifiers could be trained on the dataset consisting of more than 2,000 samples within only a few seconds. More importantly, the performance of the classifiers has also been improved. Considering that breast mass classification is only a special case of classification, we believe the proposed method for efficient performance improvement can also be extended to other scenarios.

The remainder of this paper will be arranged as follows. In Section 2, we will briefly revisit the related works. Then we will present the details of the developed system in Section 3. As mentioned before, we utilized deep learning models as feature extractors in DF-dRVFL. However, some adjustments have to be made to adapt the models for the classification task. The classification task is implemented by a novel classifier called deep random vector functional link network (dRVFL). For comparison, we also take machine learning models, including ELM, RVFLN, and spiking neural network (SNN), as the classifiers. We will present the details of these machine learning models and examine the overall classification performance of these models, where the results will be shown in Section 4. At the beginning of the experiment section, we will introduce the details of the dataset used in this chapter. We then move to parameter settings, followed by the ablation for model refinement. The experiment results will be shown in the last part of this section. We then discuss some related issues in Section 5 and end this paper with the conclusion and future work in Section 6. The abbreviations used in this paper are listed in Table 1.

**Table 1:** Symbols and meaning

Abbreviation	Definition
*CNN*	Convolutional neural network
*CAD*	Computer-aided system
*CADe*	Computer-aided detection system
*CADx*	Computer-aided diagnosis system
*DDSM*	Digital Database for Screening Mammography [12, 24]
*AUC*	Area under the ROC Curve
*ROI*	Region of Interest
*ELM*	Extreme learning machine
*RVFLN*	Random Vector Functional Link Networks
*dRVFL*	deep random vector functional link network
$DF-dRVFL$	deep feature-based deep random vector functional link network
*SNN*	Spiking neural network
*SVM*	Support vector machine
*WARM*	Weighted association rule mining
$mini-MIAS$	Mammographic Image Analysis Society [49]
*GLRM*	Gray-level run-Length matrix
*GLCM*	Gray-level co-occurrence matrix
*RNN*	Recurrent neural network
*MCC*	Matthews Correlation Coefficient
*DrpoL*	Dropout layer
*FCX*	Fully connected layer
*FFNN*	Feed-forward neural networks
*SHFN*	Single hidden layer feed-forward neural network
*SGDM*	Stochastic Gradient Descent with Momentum
*KNN*	K nearest neighbor
*DT*	Decision tree
*BDT*	Bagged Decision Tree
*ANN*	Artificial Neural Networks

## Related works

Breast mass can be generally classified into two categories, benign and malignant mass. The main difference between these two kinds of masses is that malignant mass is cancerous and may lead to death if no timely treatment is applied, while benign mass is milder and non-cancerous. Mammography is a useful modality for diagnosing breast mass. It helps doctors to diagnose breast mass in the early stage and gives medications to the patients to avoid death [31, 54]. Before the CAD systems were proposed, only well-trained radiologists and doctors could manually classify breast masses. However, traditional CAD systems include multiple steps such as pre-processing, segmentation, feature extraction, feature selection, and final classification [19]. Compared with deep learning-based CAD systems, traditional CAD systems are narrow and brittle [36]. In contrast, deep learning-based methods integrate multiple modules into deep convolutional networks with higher performance. Considering the advantages of deep learning-based methods, we will mainly review the related works implemented via deep learning. Moreover, there are also some works related to discriminative feature learning which are highly related to our work [4, 48].

Since deep learning has achieved great success in many domains, more researchers are try to integrate the deep learning method into CAD systems. For instance, Dhahri et al. [5] used the Tabu search to extract the feature maps, then applied the K-Nearest Neighbors algorithm to classify the breast lesions. The proposed method evaluates on the BIDMC-MGH dataset with an AUC of 95% and the WDBC dataset with an accuracy of 98.24%. Li et al. [26] proposed a DenseNet-II model to classify benign and malignant mammograms, and their model is based on the DenseNet with modifications. It tests on the dataset from the First Hospital of Shanxi Medical University and gets an average accuracy of 94.55%. Zhang et al. [55] designed a novel model based on feature fusion for mass classification. This model was evaluated on the CBIS-DDSM dataset and got a receiver operating curve value of 0.97, an accuracy of 94.30%, and a specificity of 97.19%. Another work using the GAN-based method was proposed by Muramatsu [32]. In this work, they used additional synthetic data from the cycle GAN and evaluated on the DDSM dataset with an accuracy of 81.4%. Khan et al. [20] implemented a multi-view feature fusion to improve the performance of CNN further to classify the malignant and benign. Finally, the proposed method reached an AUC of 0.84 on the CBIS-DDSM dataset.

There are some works based on transfer learning. In work [25], the authors proposed to apply transfer learning based on AlexNet for breast mass classification. To validate the effectiveness of mass context to the overall performance of the deep learning models, the authors extracted breast mass patches that were marginally larger than the breast mass and patches that were two times of the mass bounding box. There are different data augmentation methods, including rotation, cropping, and flipping, were applied to augment the size of the training set by a factor of 25. These data augmentation methods can alleviate problems with data scarcity. The dataset comprised 1820 images from DDSM, of which 80% were partitioned into the training set, and the remaining 20% of the images were randomly yet evenly partitioned into the validation set and testing set, respectively. The model based on GoogLeNet, however, showed the best performance that provided an accuracy of 0.929. Similar conclusions were drawn by the authors of the work [17]. In another AlexNet-based work [41], the authors proposed to transfer AlexNet for feature extraction while taking a supporting vector machine (SVM) as the classifier. The experiments on the DDSM dataset showed that the accuracy of the transferred AlexNet was only 71.01%, while the accuracy increased to 79% when SVM was fed with the features extracted by the transferred AlexNet. Another work compared the performance of multiple different models, including AlexNet, VGG16, ResNet50, InceptionV3, and DenseNet-121 on DDSM in [23]. It was concluded that VGG16 performed best with an area under the curve (AUC) of 0.82 when no data augmentation was applied. DenseNet121, however, turned out to be the best performing with an AUC of 0.91 when data augmentation was applied. Ensemble learning is another widely applied technique that aims at improving classification performance by combining the classification results from multiple models. Compared to single model-based methods, ensemble learning-based methods enjoy higher stability and robustness. In work [42], the authors proposed to ensemble AlexNet-based models for better classification performance. The three best models with the best results were selected, and the conclusion was based on the average probability. Experiments showed that the performance of individual models ranged from 75% to 77%, while the combined result was over 80%.

While some of the single-stage methods rely heavily on human intervention, multiple-stage methods that require less human intervention are more in demand and popular. Compared to single-stage methods, multiple-staged methods aim at extracting useful features in the early stages that may benefit the classification in the later stage. In work [19], authors developed a novel breast mass classification method based on weighted association rule mining (WARM). Initially, mammograms were pre-processed for contrast enhancement while the pectoral muscle was removed. In the processed mammograms, each mammogram was divided into the non-overlapping block, within which the sum average feature was calculated. The block with highest sum average feature was selected as the seed of the region growing for breast mass segmentation. By doing so, the breast mass patch can then be extracted. The latent rules between the extracted patches and the targeted classes were valued. The patch was classified as benign if the rules were more inclined to benign and vice versa. The experiment on Mammographic Image Analysis Society (mini-MIAS) showed that the proposed method achieved 95.15% accuracy on the testing set, which outperformed other methods significantly. Another work that integrated breast mass segmentation and classification can be found in [40]. In this work, a novel breast mass segmentation method called adaptive fuzzy C-means clustering was introduced. Two feature extraction methods, gray-level run-Length matrix (GLRM) and gray-level co-occurrence matrix (GLCM) are deployed to extract features from the extracted patches. Finally, the extracted features were classified by a recurrent neural network (RNN) that was optimized by a novel optimization algorithm called Average Fitness New Updating-based GrassHopper. The proposed method was evaluated on mini-MIAS. In terms of segmentation accuracy, sensitivity, $F1_{score}$, and Matthews Correlation Coefficient (MCC), the proposed method turns out to be the best compared to other state-of-the-art methods. For the classification task, the authors compared the proposed method with traditional machine learning techniques such as decision tree, SVM as well as deep convolutional neural network DCNN model. The experiment results showed that the proposed method showed the best performance against other methods from the perspective of almost all evaluation metrics.

**Table 2:** Related works

Author	Method	Results
Dhahri et al. [5]	Tabu search and K-Nearest Neighbors algorithm	An AUC of 95% for the BIDMC-MGH dataset and an accuracy of 98.24% for the WDBC dataset.
Li et al. [26]	DenseNet-II	An average accuracy of 94.55% on the dataset from the First Hospital of Shanxi Medical University.
Zhang et al. [55]	feature fusion	A receiver operating curve value of 0.97, an accuracy of 94.30%, and a specificity of 97.19% on the CBIS-DDSM dataset.
Muramatsu [32]	GAN-based method	An accuracy of 81.4% with the DDSM dataset.
Khan et al. [20]	multi-view feature fusion	An AUC of 0.84 on the CBIS-DDSM dataset.
Levy et al. [25]	transfer learning based on AlexNet	An accuracy of 0.929 on the DDSM dataset.
Ragab et al. [41]	transfer learning based on AlexNet and SVM	An accuracy of 79% on the DDSM dataset.
Kulkarni and Rabidas [23]	DenseNet121 with data augmentation	An AUC of 0.91 on the DDSM dataset.
Rampun et al. [42]	Ensemble-based method	Over 80% classification accuracy and area under the curve on the CBIS-DDSM dataset.
Keyvanpour et al. [19]	weighted association rule mining-based method	An accuracy of 95.15% on the Mammographic Image Analysis Society.
Patil et al. [40]	adaptive fuzzy C-means clustering	An accuracy of 0.96, a sensitivity of 0.96, a specificity of 0.97, and a precision of 0.92 on the MIAS Mammography dataset.

We conclude these works in Table 2, as can be seen from the mentioned methods. Some works were evaluated on the testing set with small sizes, and only tens or thousands of images were evaluated. Another issue is the performance of existing methods. Despite the fact that numerous deep learning models may show different performances regarding the classification task and the best model can be chosen for the task. It remains a problem with performance improvement at a minimal cost. Therefore, we developed a novel hybrid framework DF-dRVFL for breast mass classification in this work.

## Methodology

In this work, the developed DF-dRVFL mainly consists of two modules including, a deep feature extractor VGG19-DF and a classifier dRVFL. Initially, we use different deep learning models by transfer learning as the backbone to classify the breast mass images, and these deep learning models are VGG19, ResNet101, InceptionV3, DenseNet201, and IncepresNetv2 [11, 14, 47, 50, 51]. After comparing the performance of these transfer learning-based deep learning models, we chose the VGG19 with the best performance as the backbone of our new feature extractor called VGG19-DF. After choosing the deep learning models, we designed a novel classifier called dRVFL to use the feature maps generated from the VGG19-DF to get better performance compared with the original classifier. To evaluate the performance of our proposed classifier, we also test different classifiers, including ELM, RVFLN, and SNN on the same dataset for comparison. In the remaining section, we will illustrate the details of VGG19-DF in Section 3.1 and express the process of choosing the best classifier in Section 3.2.

### VGG19-DF: deep feature extractor

**Fig. 1:**
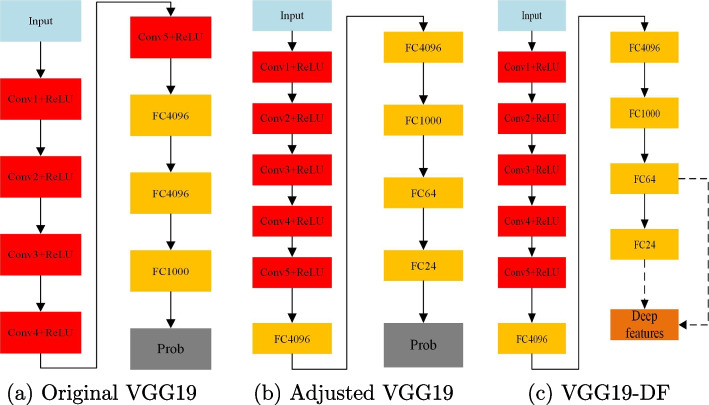
The architecture of the feature extractor before and after adjustment. The dashed lines in Fig. 1c mean the possible path to obtain deep features

To construct the deep feature extractors for the following classification task, we deployed transfer learning for efficient model acquisition. We first adjusted the architectures of the pre-trained deep learning models by removing the top layers that were initially designed for the 1000-class classification task. On the top of the fully connected layer for 1000-class classification, we added several stacked layers, including two dropout layers and three fully connected layers. If we denote the dropout layer as DropL and the fully connected layer with X neurons as FCX (For example, FC24 means a fully connected layer with 24 neurons, and FC64 is a fully connected layer with 64 neurons), the architecture of the adjusted top layers can be represented as FC1000-DropL-FC64-DropL-FC24-FC2-Prob, where Prob stands for the classification layer that takes softmax as the activation function. Note that a large dropout probability may slow the convergence. Therefore, we set the dropout probability to 0.3 for faster convergence. The architecture of the deep feature extractor based on VGG19 can be seen in Fig. 1. The adjusted deep models are then trained with the training set for feature learning, while deep features can then be extracted from the trained models. Note that deep features can be obtained from different depths of the deep learning models. Empirically, we then extract the features from the fully connected layers, as shown in Fig. 1c, and more details will be presented in the experiment section.

### Design of classifiers

The second component of DF-dRVFL is a classifier called dRVFL. The choice of the dRVFL is based on the exploration of performance by the multiple classifiers on the breast mass classification tasks. Instead of deploying traditional feature classifiers such as decision tree (DT) and SVM, we proposed four classifiers based on the architectures and algorithms behind these novel classifiers: ELM, RVFLN, dRVFL, and SNN. By the comparison of the performance of these classifiers, we found that dRVFL based model achieved the best performance. Therefore, we named the developed model DF-dRVFL.

#### ELM classifier

The proposal of the ELM algorithm aims to solve the slow training problem with traditional feed-forward neural networks (FFNN). The slow training problem can be tracked back to the iterative training due to the gradient-based learning algorithms. Instead of training the networks via iterative training, ELM randomly chooses the nodes in the hidden layer of the single hidden layer feed-forward neural networks (SHFN) and then determines the output weights via analysis. By doing so, the training time has been significantly reduced while providing good generalization performance, although the architectures of the neural networks remain unchanged.

Considering a series of observed samples *X* and the desired output *Y* that can be denoted as $\left( {\textbf {x}}_{i},{\textbf {y}}_{i}\right)$, so that ${\textbf {x}}_{i}=\left[ x_{i1},\cdots ,x_{iu}\right] ^{T} \in \mathbb {R}^{u}$ and ${\textbf {y}}_{i}=\left[ y_{i1},\cdots ,y_{iv}\right] ^{T} \in \mathbb {R}^{v}$. If we denote the number of the observations as *N*, the activation function as *s*(*x*), and the number of hidden nodes as *h*, then the output ${\textbf {O}}$ can be modeled by

1 $$\begin{aligned} {\textbf {o}}_{j}=\sum _{i=1}{h}\alpha _{i}s({\textbf {w}}_{i}\cdot {\textbf {x}}_{j}+th_{i}) \end{aligned}$$

where ${\textbf {o}}_{j}$ is the output of *j*th node in the output layer, $j=1,\cdots ,N$, ${\textbf {w}}_{i}=\left[ w_{i1},\cdots ,w_{iu}\right] ^{T}$ is the weight vector between the input nodes and *i*th hidden node. $\alpha _{i}=\left[ \alpha _{i1},\cdots ,\alpha _{im}\right] ^{T}$ and $th_{i}$ stands for the threshold value of *i*th hidden node. The operation $\cdot$ means the inner product of ${\textbf {w}}_{i}$ and ${\textbf {x}}_{i}$. Ideally, the SHFN can approximate the expected output of these *N* samples with zero means by

2 $$\begin{aligned} {\textbf {y}}_{j}=\sum _{i=1}{h}\alpha _{i}s({\textbf {w}}_{i}\cdot {\textbf {x}}_{j}+th_{i}) \end{aligned}$$

The (2) can also be written as ${\textbf {Y}}={\textbf {B}}\alpha$, where **B** is called the hidden layer output matrix. The corresponding cost function can be expressed as

3 $$\begin{aligned} E=\sum _{j=1}^{N}\left( \sum _{i=1}^{h}\alpha _{i}s({\textbf {w}}_{i}\cdot {\textbf {x}}_{j}+th_{i})-{\textbf {y}}_{j}\right) ^2 \end{aligned}$$

**Fig. 2:**
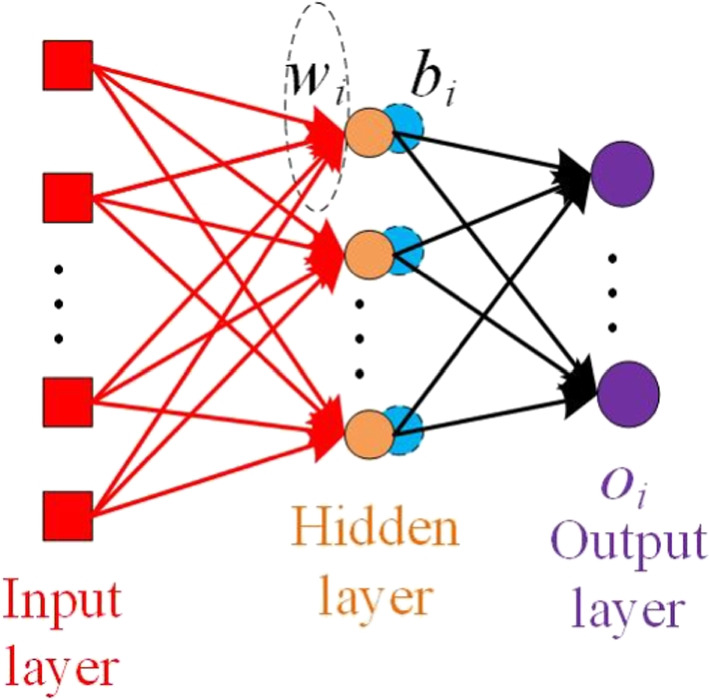
The architecture of extreme learning machine

To minimize the error from gradient-based algorithms, the parameters including ${\textbf {W}}$, which is the vector form of ${\textbf {w}}_{i}$, $\varvec{\alpha }$, and ${\textbf {b}}$ will be iteratively updated. The iterative updating of ${\textbf {W}}$ can be denoted as:

4 $$\begin{aligned} {\textbf {W}}_{n}={\textbf {W}}_{n-1}-\beta \frac{\partial E({\textbf {W}})}{\partial {\textbf {W}}} \end{aligned}$$

where $\beta$ here is the learning rate. Back propagation is usually used as the learning algorithm so that errors can be back forwarded for parameter optimization. However, some issues on the back propagation appeared. The first one is the definition of $\beta$. If it was too small, it took much longer time for the learning algorithm to converge. However, a large $\beta$ may lead to instability or even divergence. Local minima and the time-consuming gradient-based learning are other perplexing issues. The difference between algorithm ELM and back propagation mainly lies in the method for parameter updating. The main procedures of ELM can be seen in Algorithm 1. As can be seen, there are just several matrix multiplication operations with the algorithm, so the training time is much less than the gradient-based algorithms. The architecture of ELM is shown in Fig. 2. For ELM, the number of neurons in the hidden layer is the key factor that determines the overall performance of ELM.

**Figure:**
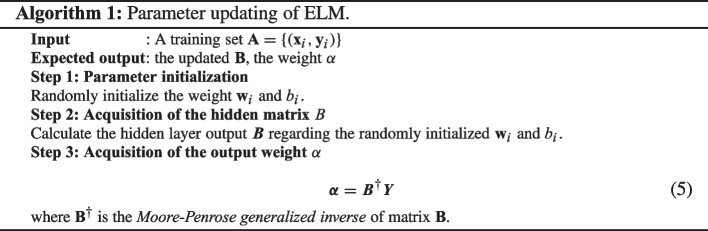
Parameter updating of ELM.

#### RVFLN classifier

**Fig. 3:**
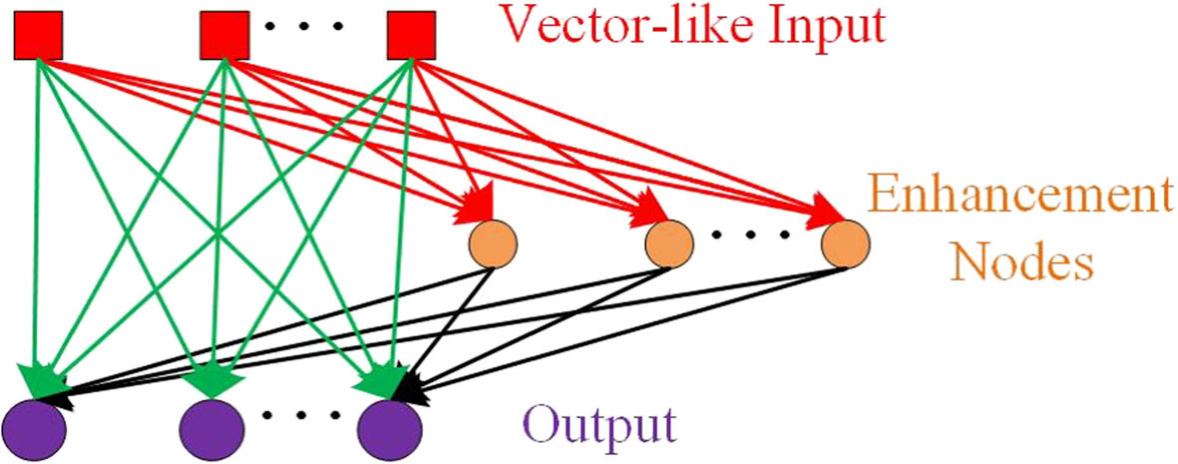
The architecture of random vector functional link net

RVFLN, which was first proposed in work [39], was another training-efficient classifier. RVFLN showed that the weights connecting the input layer with the hidden layer could be randomly generated while these generated weights can be fixed during the training stage. Also, on the bounded finite dimensional set, RVFLN has been proven to be a universal approximator for a continuous function with a close-form solution [15]. The architecture of RVFLN can be seen in Fig. 3. The neurons between the input layer and the output layer are called enhancement nodes.

As can be seen, the output layer receives both the original input ${\textbf {Y}}$ from the original input layer and the transformed features ${\textbf {H}}$ from the hidden layer. Like ELM, the weights connecting the hidden layer with the output layer are randomly generated and are fixed during the training stage. Only output weights $\alpha _{s}$ need to compute so that the optimization problem can be represented as:

6 $$\begin{aligned} Obj=\mathop {min}\limits _{\alpha _{s}}||B\alpha _{s}-Y||^{2}+\eta ||\alpha _{s}||^{2} \end{aligned}$$

where ${\textbf {B}}=\left[ H X\right]$, which is the concatenated features from the input layer and the hidden layer. $\eta$ is the regularization parameter. The (6) can be solved via either Moore-Penrose pseudoinverse (when $\eta =0$) or ridge regression (when $\eta \ne 0$). If the Moore-Penrose pseudoinverse is used, the solution will be (5) in Algorithm 1. When ridge regression is used, the close-form solution will be $\alpha _{s}=({\textbf {B}}^{T}{} {\textbf {B}}+\eta {\textbf {I}})^{-1}{} {\textbf {B}}^{T}{} {\textbf {Y}}$ in the primal space or $\alpha _{s}={\textbf {B}}^{T}({\textbf {B}}^{T}{} {\textbf {B}}+\eta {\textbf {I}})^{-1}{} {\textbf {Y}}$ in the dual space. The main computational cost comes from the matrix inversion operation, which can be avoided using either a primal or dual solution.

#### dRVFLN classifier

**Fig. 4:**
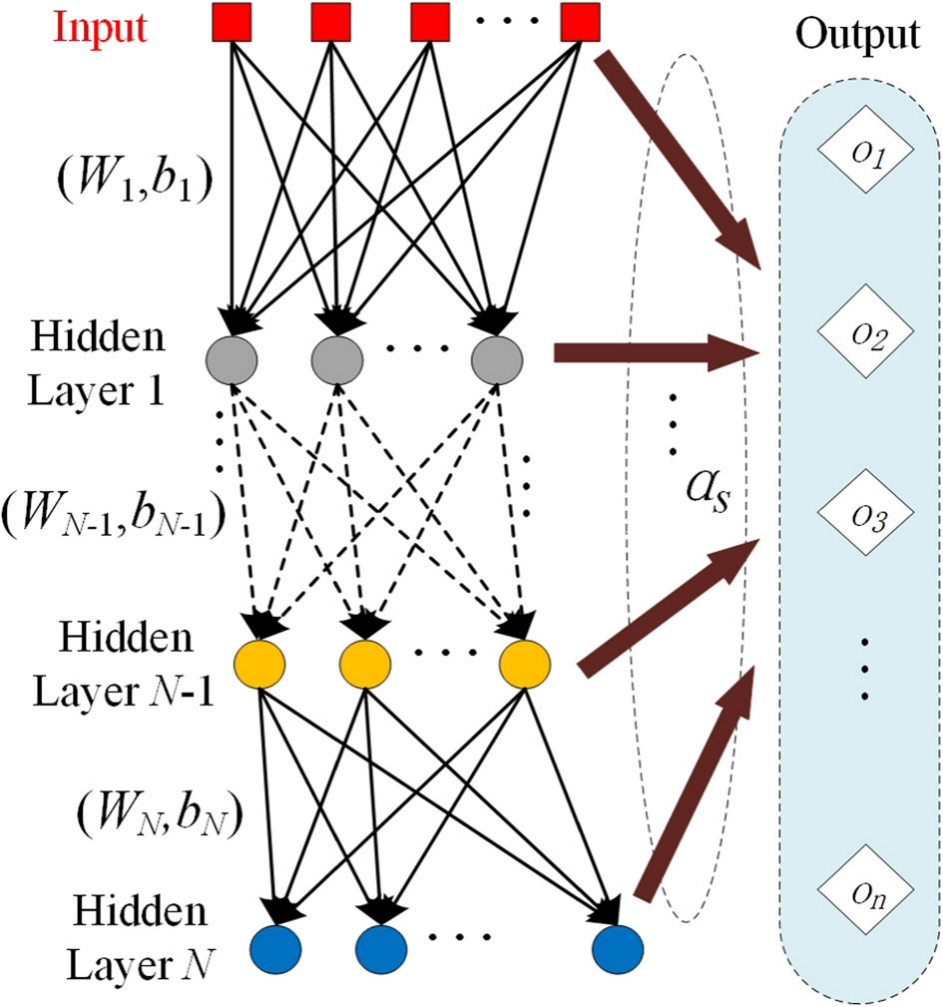
The architecture of deep random vector functional link net

Based on the original RVFLN, we proposed to employ an improved version of RVFLN called dRVFL in [46] as the classifier. In dRVFL, there are usually several stacked hidden layers between the input layer and the output layer. Like RVFLN, the input is concatenated with the output of the last hidden before the concatenated features go into the output layer. The weights between the input layer and the first hidden layers, as well as the weights between hidden layers are randomly generated. By doing so, the optimization process is much more efficient compared to the back propagation-based optimization pattern. The architecture of dRVFL can be seen in Fig. 4.

Given the input **X**, the activation function for each hidden layer as $s(\cdot )$, and no bias term considered, the output of the first hidden layer can be denoted as:

7 $$\begin{aligned} {\textbf {H}}^{1}=s({\textbf {XW}}_{1}) \end{aligned}$$

Similarly, the output of the *L*th hidden layer can be denoted as:

8 $$\begin{aligned} {\textbf {H}}^{L}=s({\textbf {H}}^{L-1}{} {\textbf {W}}_{L}) \end{aligned}$$

where $\varvec{W}_{1}$ and $\varvec{W}_{L}$ are the weight matrices between the input and first hidden layer and the $L-1$th hidden layer and the *L*th hidden layer, respectively. Similar to parameters in ELM, these parameters are randomly generated and will not be updated during the training session. Then the input features fed to the output layer can be expressed as:

9 $$\begin{aligned} \varvec{B}=\left[ \varvec{H}^{1} \cdots \varvec{H}^{L} \varvec{X}\right] \end{aligned}$$

The output can then be obtained via

10 $$\begin{aligned} \varvec{Y}=\varvec{B}\varvec{\alpha }_{s} \end{aligned}$$

So that $\varvec{\alpha }_{s}$ can be calculated via Moore-Penrose pseudoinverse as was shown in (5).

#### SNN classifier

The SNNs were first proposed by Maass [29]. SNN is a type of ANNs that simulates the behavior of biological neurons. They are characterized by their ability to model the dynamics of neural firing through the use of spike-time coding, which is based on the timing of individual action potentials rather than their frequency. Due to these features, the SNNs can integrate information from different aspects such as time, frequency, and phase [8, 13, 16, 18, 52]. There are some works about deploying SNNs in the computer vision tasks. Masquelier and Thorpe [30] use SNNs to model the behavior of the visual cortex in response to naturalistic visual stimuli. Kheradpisheh et al. [21] designed a SCNN to extract the edges, and Panda and Roy [38] suggested a convolutional Auto-Encoder learning method for SNN. Moreover, an SNN-based object detection system Spiking-YOLO was developed that converges 2.3 to 4 times faster than the previous SNN methods [22]. Another study demonstrated the effectiveness of using SNNs for gesture recognition, achieving accuracy comparable to that of traditional neural networks while using significantly less energy [45]. Overall, SNNs offer a promising approach to performing efficient and energy-efficient event-driven processing of spatiotemporal information in computer vision tasks.

In our framework, we design the SNN model by adjusting the model presented in work [37]. Initially, this work was evaluated on the MNIST dataset by vectorizing the images into the vectors. In our classification task, we, instead, proposed to feed the model with the features extracted by our trained deep feature extractors.

**Fig. 5:**
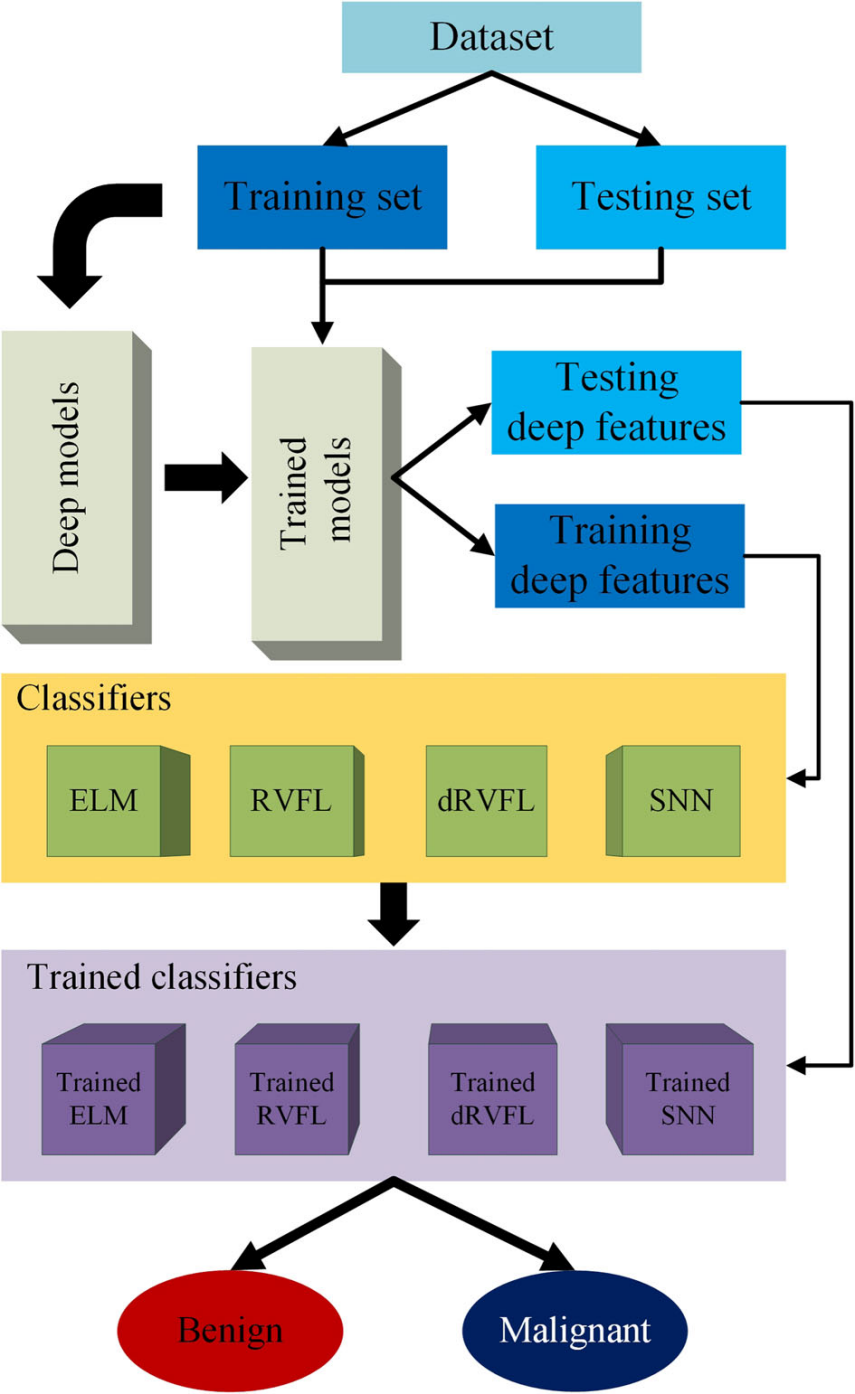
The overall flowchart of the DF-dRVFL

The overall flow chart for model training is shown in Fig. 5.

## Experiment

In this section, we will mainly introduce the experiment-related issues of this study. To begin with, we will briefly introduce the information of the dataset used in this study, followed by the settings of the experiment. By saying the settings, we mean the settings, including hardware configurations, parameter settings, and so forth. Subsequently, we will compare the performance of the feature extractors by evaluating the classification performance of these deep CNN models on the classification task. After we determined the feature extractor for the following classifiers, we then explored the optimal architectures of the classifiers to obtain the best-performed model via the ablation experiment. Finally, we will compare our method with traditional machine learning-based methods and state-of-the-art methods at the end of the section.

### Dataset

The images involved in this study for method training and evaluation come from the DDSM dataset [12, 24]. Note that we simply aimed to evaluate the performance of the developed breast mass classification system here. Therefore, manually cropped ROI from the full field mammograms. We used five-cross validation to train and evaluate the models on the mass-level dataset of the DDSM, which can be found at https://www.kaggle.com/datasets/skooch/ddsm-mammography. The detailed information of each randomly partitioned subset can be seen in Table 3. As can be seen, the number of benign masses and malignant masses are roughly the same.

**Table 3:** Patch-level dataset from DDSM for model training and validation

	1	2	3	4	5	Total
Benign	382	381	382	381	382	1908
Malignant	362	362	362	362	362	1810
Total	744	743	744	743	744	3718

### Settings of the experiment

In this section, we will mainly introduce the configurations of the experiment, including the hardware environment, the hyper-parameter setting for the feature extractor, and the architectural settings of the feature classifiers. For hardware configuration, we deployed the SPECTRE High-Performance Computing Facility provided by the University of Leicester for model training and validation. The memory of the facility is 16 GB. For the deep learning models trained for the breast mass classification task, we used the parameters listed in Table 4. Another factor that affects the overall classification performance is the architectures of the classifiers, such as dRVFL, as it was known that both ELM and RVFLN have only one hidden layer. For dRVFL, we set the number of layers to be three by default because deeper networks are more likely to be overfitted. As for the number of neurons in the classifiers, we will determine them via ablation experiments.

**Table 4:** The setting of hyper-parameters for deep models

Parameters	Values
Maximum training epoch	12
Initial learning rate	$5*10^{-4}$
Mini-batch size	60
Learning rate drop period	3
Learning rate drop rate	0.1
Optimization method	SGDM
Shuffle of the train set	Each epoch

### Performance of feature extractors

In this study, we employed state-of-the-art deep learning models, including VGG19, ResNet101, InceptionV3, DenseNet201, and IncepresNetv2 [11, 14, 47, 50, 51]. The performance of these deep models is mainly determined by the number of learnable parameters and the architectures, while the architectures of these models can be indicated by the number of connections between the layers. We then listed the numbers of the parameters and the numbers of the connections in Table 5, where we use $Rate_{CL}$, which is the rate between the number of connections to the number of layers, to show the architectural complexity of the deep models. As can be seen, VGG19 turns out to be the most complicated model in terms of the number of learnable parameters. DenseNet201, however, is the most complicated if we consider the $Rate_{CL}$, while VGG19 becomes the simplest one.

**Table 5:** Architectural details of deep models

Model names	Number of learnable	Number of layers	Number of	$Rate_{CL}$
	parameters (Millions)		connections	
VGG19	**143.9**	52	51	0.98
ResNet101	44.6	351	383	1.09
InceptionV3	23.9	319	353	1.11
DenseNet201	20.0	713	810	**1.14**
InceptionResNetv2	55.9	829	**926**	1.12

**Fig. 6:**
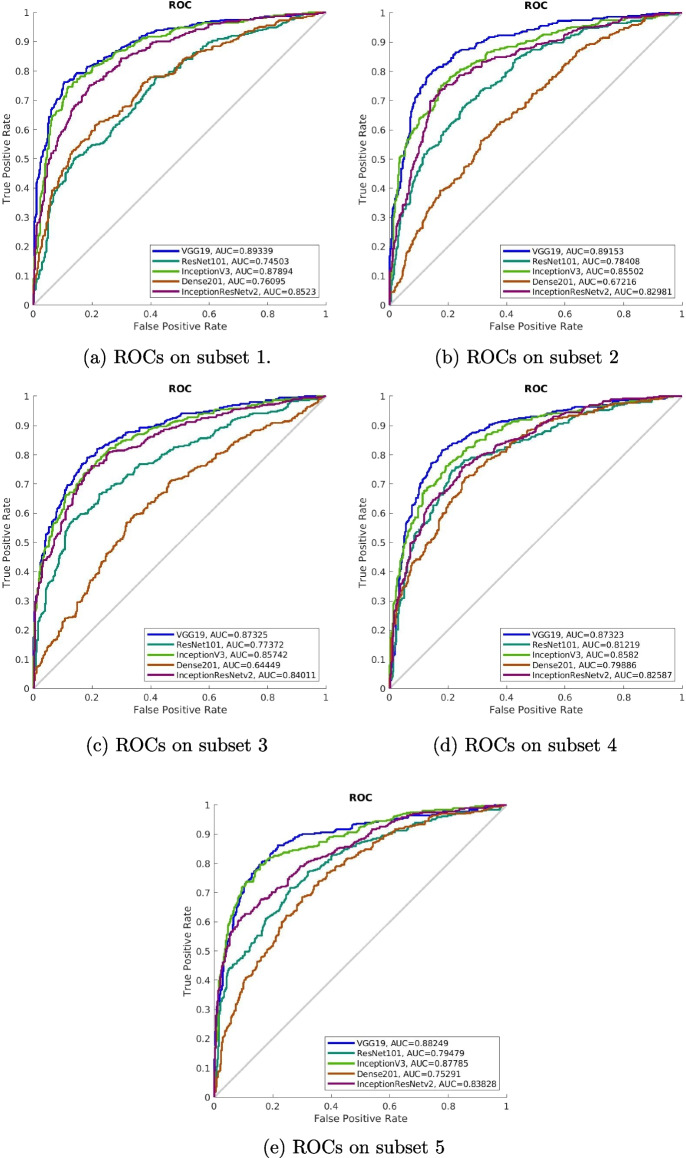
ROCs of deep models on DDSM

To evaluate the performance of the trained deep models, we used the same evaluation metrics, including sensitivity, specificity, accuracy, precision, $F1_{score}$, and AUC. As mentioned before, we carried out the five cross-validation on DDSM and calculated the averaged evaluation metrics regarding the deep learning models trained five times individually. The results are shown in Table 6. The corresponding ROCs can be seen in Fig. 6.

**Table 6:** Performance of the deep learning models toward breast mass classification

Models	Sensitivity	Specificity	Precision	$F1_{score}$	Accuracy	AUC
VGG19	**81.19±1.66**	** 81.81±3.76**	**82.97±5.01**	** 81.97±1.87**	**81.33±1.29**	**88.28±0.96**
ResNet101	$70.27\pm 3.47$	$72.27\pm 3.28$	$76.05\pm 2.58$	$73.03\pm 2.86$	$71.14\pm 3.35$	$78.20\pm 2.51$
InceptionV3	$78.49\pm 2.06$	$80.56\pm 1.50$	$82.70\pm 1.14$	$80.54\pm 1.59$	$79.48\pm 1.80$	$86.55\pm 1.18$
DenseNet201	$64.42\pm 5.81$	$68.34\pm 4.92$	$76.68\pm 1.95$	$69.88\pm 3.51$	$65.87\pm 5.71$	$72.59\pm 6.48$
InceptionResNetv2	$76.07 \pm 1.46$	$76.79\pm 1.95$	$78.77\pm 2.34$	$77.39\pm 1.50$	$76.38\pm 1.44$	$83.73\pm 1.03$

As can be seen from the above table and figures, VGG19 performed best among all of the models in terms of sensitivity, accuracy, AUC, and other metrics. Therefore, we believe VGG19 is more suitable to be the feature extractor and performance extraction for the following feature classifiers. Note that the AUCs of VGG19 are stable, but they varied slightly around 0.88. VGG19 may benefit from the large volume of the number of parameters and the straightforward architecture. However, the number of learnable parameters is not the only factor that determines the overall classification performance. As can be seen, InceptionV3 has fewer parameters compared to ResNet101. However, the overall performance of InceptionV3 greatly outperformed the ResNet101. Nevertheless, the conclusion here doesn’t deny the high performance of the other deep models, but more fine-tuning procedures should be introduced for better performance. For the experiment, we take the trained VGG19 model as the the backbone of our feature extractor VGG19-DF.

### Model ablation

Although we have successfully trained the deep models for feature extraction, the final performance of the classifiers, however, not only depends on the performance of feature extraction but also relies on utilizing the feature extractor for feature extraction and the architectures of the following classifiers. In this section, we will carry out the ablation experiment to specify the best configurations of the feature classifiers and the integrations of the feature extractor and feature classifiers. The output size of the feature extractor can vary from 1000 to 2. However, a large size of the output will unnecessarily increase the computational cost, while a small size of output suffers from significant information loss. Therefore, we take the deep feature output from the FC24 layer and FC64 as the features for the classifiers. For simplicity, we denote the feature representation generated from FC24 as $Fea_{24}$ and the feature representation generated from FC64 as $Fea_{64}$. By concatenating $Fea_{24}$ and $Fea_{64}$, then the concatenated feature map is $Fea_{24+64}$. Inspired by the work in [28], we believe the classifiers may gain extra benefit by learning from multiple-level features. We then consider the performance of the classifiers under these three situations.

#### $Fea_{24}$-based mass classification

We first explored the performance of the classifiers, i.e., ELM, RVFLN, and dRVFL. To specify the most optimal number of the hidden layer, we then varied the number from 40 to 1,000 and concluded the results in Table 7. As can be seen from Table 7, the ELM model with 1000 hidden nodes gives the highest overall accuracy compared to ELMs with other numbers of hidden neurons. However, the highest AUC is given by the ELM model with only 40 or 80 hidden nodes. Also, these models have very close performance to the performance of the trained VGG19 models, while some of them showed even worse performance. Therefore, it may not be suitable to take ELMs as the feature classifiers.

**Table 7:** Performance of the extreme learning machine with the varied numbers of hidden neurons based on deep feature $Fea_{24}$(unit:%)

Number of	Sensitivity	Specificity	Precision	$F1_{score}$	Accuracy	AUC
hidden						
neurons						
40	79.01±2.09	**83.18±2.44**	**81.73±1.94**	80.31±0.85	81.15±0.82	86.14±1.08
80	79.83±1.31	82.55±2.54	81.33±2.05	80.55±0.92	81.23±1.09	86.14±1.01
100	79.72±1.62	82.55±2.62	81.30±2.20	80.48±1.25	81.17±1.35	85.70±1.22
200	79.94±1.59	82.70±2.21	81.47±1.84	80.68±1.09	81.36±1.13	**85.84±0.88**
400	79.83±1.14	82.70±2.35	81.45±2.02	80.62±1.08	81.31±1.20	85.31±1.10
800	79.83±0.81	82.55±1.79	81.29±1.58	80.55±0.90	81.23±1.00	85.35±1.24
1000	**80.28±1.42**	82.86±1.83	81.65±1.62	**80.95±1.18**	**81.60±1.18**	85.58±1.22

For RVFLN, the number of enhancement nodes controls the overall performance of the RVFLN models. Similarly, we then varied the number of enhancement nodes to determine the best model, and the results can be seen in Table 8. As can be seen, the RVFLN model with 100 enhancement nodes is the best model compared to RVFLNs with other numbers of enhancement nodes in terms of precision, $F1_{score}$, and accuracy. Generally, all RVFLN models showed competitive performance against each other while showing higher performance than ELM when the input feature is $Fea_{24}$. Besides, the RVFLN models with different numbers of enhancement neurons also showed close or even better performance against the VGG19 models. Compared to the previous ELM models, RVFLN showed better performance, so we believe RVFLN models are preferable to be the classifiers for breast mass classification tasks.

**Table 8:** Performance of the random vector functional link with the varied number of enhancement nodes based on deep feature $Fea_{24}$(unit:%)

Number of	Sensitivity	Specificity	Precision	$F1_{score}$	Accuracy	AUC
hidden						
neurons						
40	$79.28\pm 1.71$	$83.23\pm 2.88$	$81.84\pm 2.38$	$80.51\pm 1.10$	$81.31\pm 1.24$	$89.01\pm 0.94$
80	$79.61\pm 1.48$	$83.12\pm 3.28$	$81.83\pm 2.69$	$80.67\pm 0.98$	$81.41\pm 1.27$	$89.01\pm 0.96$
100	$79.50\pm 1.83$	${\textbf {83.28}}\pm {\textbf {3.14}}$	${\textbf {81.95}}\pm {\textbf {2.51}}$	80.67±0.84	${\textbf {81.44}}\pm {\textbf {1.06}}$	$89.06\pm 0.96$
200	$79.50\pm 2.43$	$83.23\pm 3.21$	$81.91\pm 2.53$	$80.64\pm 1.12$	$81.41\pm 1.18$	$89.05\pm 0.95$
400	${\textbf {79.94}}\pm {\textbf {2.52}}$	$82.70\pm 3.56$	$81.55\pm 2.74$	${\textbf {80.68}}\pm {\textbf {1.02}}$	$81.36\pm 1.16$	${\textbf {89.07}}\pm {\textbf {0.92}}$
800	$79.83\pm 2.45$	$82.76\pm 3.50$	$81.57\pm 2.72$	$80.64\pm 1.03$	$81.33\pm 1.18$	$89.01\pm 0.96$
1000	$79.83\pm 2.21$	$82.70\pm 3.45$	$81.52\pm 2.73$	$80.62\pm 1.09$	$81.31\pm 1.27$	$89.06\pm 0.96$

For dDVFL, the number of hidden layers and the number of neurons within each layer of the dRVFL models collaboratively determine the final performance. As we decided to set the number of layers to three, we then varied the number of neurons within each hidden layer. Also, we keep the number of hidden neurons in each layer the same. To avoid overfitting issues, we set the number of hidden neurons to be relatively small as we range the value from 6 to 24 with an interval of 6. The results can be seen in Table 9. As can be seen, the dRVFL model with six enhancement nodes turns out to be the best model compared to dRVFL models with other numbers of enhancement nodes in terms of sensitivity, accuracy, and AUC. Note that the average accuracy has been improved to $81.60\%$. Also, compared to ELMs and dRVFL models, dRVFL models showed consistent performance on DDSM while possessing higher AUCs, which indicated the suitability of these models to be utilized as classifiers.

**Table 9:** Performance of the deep random vector functional link nets with the varied number of enhancement nodes based on deep feature $Fea_{24}$(unit:%)

Number of	Sensitivity	Specificity	Precision	$F1_{score}$	Accuracy	AUC
enhancement						
neurons						
6	**83.07±3.01**	$80.06\pm 2.01$	$81.48\pm 1.11$	**82.23±1.20**	**81.60±0.93**	**93.45±1.10**
12	$82.18\pm 3.28$	**80.39±2.02**	$81.58\pm 0.96$	$81.83\pm 1.22$	$81.31\pm 0.80$	$93.09\pm 1.06$
18	$82.55\pm 4.80$	$79.78\pm 3.25$	$81.24\pm 1.57$	$81.79\pm 1.67$	$81.20\pm 1.05$	$93.33\pm 1.37$
24	$82.23\pm .4.15$	$80.39\pm 2.29$	**81.59±1.12**	$81.85\pm 1.70$	$81.33\pm 1.24$	$93.18\pm 1.23$

For SNN, the number of hidden neurons in the hidden layer determines the final performance of the SNN model. So we varied the number of hidden neurons in the hidden layer to obtain the candidate models. The results can be seen in Table 10. Note that the measurement of AUC is not applicable to the SNN models, so we only calculated the metrics, including sensitivity, specificity, precision, $F1_{score}$, and accuracy. As can be seen, the network with 40 hidden neurons turns out to be the best model compared to other SNNs with different numbers of hidden nodes in terms of accuracy. However, the overall performance of SNNs is even worse than the trained VGG19 and other classifiers. Also, the standard deviation errors of SNNs increase along with the number of hidden nodes. Therefore, SNN may not be suitable for the classification task here.

**Table 10:** Performance of the spiking neural network with the varied number of hidden nodes based on deep feature $Fea_{24}$

Number of	Sensitivity	Specificity	Precision	$F1_{score}$	Accuracy
hidden					
neurons					
40	78.40±4.11	$81.34\pm 4.76$	$79.70\pm 1.76$	${\textbf {81.24}}\pm {\textbf {1.44}}$	**80.37±1.16**
80	$77.90\pm 3.22$	$82.55\pm 4.79$	${\textbf {81.14}}\pm {\textbf {3.62}}$	$79.37\pm 0.46$	$80.29\pm 1.00$
100	$77.68\pm 5.58$	$81.76\pm 6.22$	$80.59\pm 4.39$	$78.86\pm 1.30$	$79.77\pm 1.03$
200	${\textbf {80.06}}\pm {\textbf {4.46}}$	79.35±7.16	79.08±4.95	$79.35\pm 0.76$	$79.69\pm 1.68$
400	$73.92\pm 7.04$	${\textbf {82.76}}\pm {\textbf {9.26}}$	$81.19\pm 6.73$	$76.92\pm 2.20$	$78.46\pm 2.36$
800	$75.80\pm 10.63$	$78.78\pm 13.79$	$78.83\pm 8.20$	$76.39\pm 3.05$	$77.33\pm 3.21$
1000	$70.83\pm 14.14$	$81.56\pm 16.31$	$81.09\pm 9.82$	$74.11\pm 4.38$	$76.33\pm 3.20$

#### $Fea_{64}$-based mass classification

We then explored the best models of the classifiers, i.e., ELM, RVFLN, and dRVFL based on $Fea_{64}$. Similarly, we then varied the number of hidden layers for ELM from 40 to 1,000 and concluded the results in Table 11. As can be seen from Table 11, the ELM with 80 hidden nodes performed best compared to ELMs with other numbers of hidden neurons in terms of accuracy. Compared to the ELM models that were trained on $Fea_{24}$, the models trained on $Fea_{64}$ showed higher performance. Also, the ELM models with large numbers of hidden nodes failed to perform better than the ELM models with small numbers of hidden nodes, which suggests the careful choice of the number of hidden nodes is required. Interestingly, the models trained on $Fea_{64}$ showed constantly better performance than the ELMs trained on $Fea_{64}$. Therefore, we may roughly conclude that $Fea_{64}$ is more representative than $Fea_{64}$.

**Table 11:** Performance of the extreme learning machine with the varied number of hidden neurons based on deep feature $Fea_{64}$

Number of	Sensitivity	Specificity	Precision	$F1_{score}$	Accuracy	AUC
hidden						
neurons						
40	$78.90\pm 2.13$	$82.76\pm 2.76$	$81.36\pm 2.04$	$80.06\pm 0.35$	$80.88\pm 0.49$	$85.26\pm 0.88$
80	$80.00\pm 2.07$	$83.28\pm 2.46$	$82.00\pm 2.00$	**80.96±1.14**	**81.68±1.14**	$85.63\pm 1.52$
100	**80.44±1.71**	$82.02\pm 3.08$	$81.01\pm 2.50$	$80.70\pm 1.19$	$81.25\pm 1.37$	$85.78\pm 1.25$
200	$79.78\pm 1.33$	$82.86\pm 2.73$	$81.60\pm 2.23$	$80.66\pm 0.92$	81.36$\pm 1.11$	$85.33\pm 1.38$
400	$79.45\pm 1.27$	**83.54±2.23**	**82.11±2.02**	$80.75\pm 1.28$	81.55±1.34	**85.91±1.71**
800	$80.17\pm 1.51$	$82.49\pm 2.38$	$81.34\pm 1.95$	$80.73\pm 0.98$	$81.36\pm 1.07$	$84.73\pm 1.50$
1000	$79.83\pm 1.12$	$82.49\pm 2.98$	$81.30\pm 2.57$	$80.54\pm 1.28$	$81.20\pm 1.49$	$85.05\pm 1.37$

Similarly, we then varied the number of enhancement nodes to determine the best model for RVFLN. The results can be seen in Table 12. As can be seen, the RVFLN model with 400 enhancement nodes is the best model compared to RVFLNs with other numbers of enhancement nodes. Compared to the RVFLN models that were trained on $Fea_{24}$, the RVFLN models trained on $Fea_{64}$ have a better performance than other models. This can also verify the representativeness of $Fea_{64}$.

**Table 12:** Performance of the random vector functional link with the varied number of enhancement nodes based on deep feature $Fea_{64}$

Number of	Sensitivity	Specificity	Precision	$F1_{score}$	Accuracy	AUC
hidden						
neurons						
40	$79.56\pm 2.50$	$83.02\pm 3.19$	$81.73\pm 2.53$	$80.58\pm 1.21$	$81.33\pm 1.25$	$88.83\pm 0.73$
80	$79.23\pm 2.27$	$83.44\pm 3.17$	$82.04\pm 2.58$	$80.56\pm 1.15$	$81.39\pm 1.25$	88.93±0.74
100	**79.83±2.55**	$83.01\pm 2.98$	$81.78\pm 2.31$	$80.74\pm 0.99$	$81.47\pm 0.98$	$88.90\pm 0.75$
200	$79.45\pm 2.12$	$83.38\pm 2.88$	$82.02\pm 2.32$	$80.67\pm 0.91$	$81.47\pm 1.00$	$88.92\pm 0.87$
400	$79.50\pm 2.09$	$83.44\pm 2.72$	$82.07\pm 2.20$	$80.73\pm 0.97$	**81.52±1.02**	$88.97\pm 0.94$
800	$79.67\pm 2.22$	$83.02\pm 3.00$	$81.74\pm 2.37$	$80.65\pm 0.87$	$81.39\pm 0.98$	**89.04±0.91**
1000	$79.39\pm 2.16$	**83.54±2.82**	**82.15±2.30**	**80.71±1.02**	$81.52\pm 1.08$	$88.90\pm 0.75$

For dRVFL, we varied the number of hidden neurons in each hidden layer while making sure the hidden neurons between the hidden layers were the same. The results can be seen in Table 13. As can be seen, the model with 12 neurons within three hidden layers is the best model compared to dRVFLs with other numbers of enhancement nodes. Note that the average accuracy reached $81.55\%$, which means an average difference of 0.22 between the dRVFL models and the trained VGG19s. When these trained models are compared with the models trained on $Fea_{24}$, we found that these models performed even better and should be considered as the candidate classifier.

**Table 13:** Performance of the deep random vector functional link with the varied number of enhancement nodes based on deep feature $Fea_{64}$

Number of	Sensitivity	Specificity	Precision	$F1_{score}$	Accuracy	AUC
enhancement						
neurons						
6	$82.07\pm 3.41$	$80.28\pm 2.54$	$81.48\pm 1.66$	$81.73\pm 1.64$	$81.20\pm 1.44$	$93.07\pm 1.07$
12	$81.60\pm 3.62$	**81.05±3.15**	**82.04±1.93**	81.75±1.27	$81.33\pm 0.98$	$92.96\pm 1.24$
18	$82.34\pm 3.24$	$80.44\pm 2.30$	$81.66\pm 1.27$	$81.95\pm 1.26$	$81.41\pm 0.93$	**93.24±1.07**
24	**82.60±2.51**	$80.44\pm 1.72$	$81.67\pm 1.24$	**82.11±1.39**	**81.55±1.28**	$93.10\pm 0.97$

For SNN, we then varied the number of hidden neurons for best candidate model acquisition. The corresponding results can be seen in Table 14. As can be seen, the SNN model with 40 hidden neurons is the best model compared to other SNNs with different numbers of hidden nodes. The comparison between SNN models trained on $Fea_{24}$ and on $Fea_{64}$ showed that the models trained on $Fea_{64}$ are more desirable though the overall performance of the SNN models is still worse than other classifiers.

#### $Fea_{24+64}$-based mass classification

Inspired by another work, we believe it worthwhile to concatenate the features from different levels for model training. Based on previous classifiers, we then slightly changed the architectures of those classifiers aiming at generating the best models based on the concatenated feature $Fea_{24+64}$. Similarly, we first varied the number of hidden layers for ELM from 40 to 1,000 and listed the results in Table 15. As can be seen from Table 15, the ELM model with 200 hidden neurons performed best compared to ELMs with other numbers of hidden neurons. Compared to the ELM models that were trained on $Fea_{24}$ and on $Fea_{64}$, the models trained on $Fea_{24+64}$ showed similar performance.

**Table 14:** Performance of the spiking neural network with the varied number of hidden nodes based on deep feature $Fea_{64}$

Number of	Sensitivity	Specificity	Precision	$F1_{score}$	Accuracy
hidden					
neurons					
40	$78.73\pm 2.88$	$83.49\pm 5.09$	$82.16\pm 3.99$	80.30±0.78	81.17±1.37
80	$78.73\pm 2.78$	83.28±4.37	$81.91\pm 3.31$	$80.20\pm 0.50$	$81.07\pm 0.99$
100	79.34±3.19	$83.02\pm 4.55$	$81.81\pm 3.47$	${\textbf {80.45}}\pm {\textbf {0.78}}$	${\textbf {81.23}}\pm {\textbf {1.10}}$
200	$77.73\pm 3.66$	${\textbf {83.81}}\pm {\textbf {3.78}}$	$82.17\pm 2.81$	$79.79\pm 0.70$	$80.85\pm 0.34$
400	${\textbf {79.72}}\pm {\textbf {2.77}}$	$81.60\pm 3.72$	$80.57\pm 2.59$	$80.08\pm 0.59$	$80.69\pm 0.80$
800	$76.69\pm 4.60$	$84.85\pm 4.97$	83.05±3.45	$79.58\pm 1.01$	$80.88\pm 0.74$
1000	$75.52\pm 3.17$	$85.43\pm 3.89$	${\textbf {83.30}}\pm {\textbf {3.23}}$	$79.12\pm 0.67$	$80.61\pm 0.71$

**Table 15:** Performance of the extreme learning machine with the varied number of hidden neurons based on deep feature $Fea_{24+64}$

Number of	Sensitivity	Specificity	Precision	$F1_{score}$	Accuracy	AUC
hidden						
neurons						
40	$79.94\pm 1.38$	$82.23\pm 3.28$	$81.11\pm 2.67$	$80.49\pm 1.12$	$81.12\pm 1.38$	$85.60\pm 1.06$
80	$79.50\pm 2.16$	$82.86\pm 2.95$	$81.57\pm 2.35$	$80.48\pm 1.11$	$81.23\pm 1.18$	$85.60\pm 1.08$
100	$79.17\pm 1.75$	${\textbf {83.39}}\pm {\textbf {2.17}}$	${\textbf {81.92}}\pm {\textbf {1.89}}$	$80.51\pm 1.26$	$81.33\pm 1.25$	${\textbf {86.51}}\pm {\textbf {1.39}}$
200	$79.67\pm 1.11$	$83.18\pm 1.88$	$81.82\pm 1.71$	80.72±1.13	**81.47±1.17**	$85.99\pm 1.14$
400	$80.17\pm 1.04$	$82.44\pm 2.43$	$81.29\pm 2.05$	$80.71\pm 1.00$	$81.33\pm 1.17$	$85.39\pm 1.24$
800	${\textbf {80.22}}\pm {\textbf {0.64}}$	$82.44\pm 2.71$	$81.31\pm 2.31$	${\textbf {80.75}}\pm {\textbf {1.13}}$	$81.36\pm 1.35$	$85.60\pm 1.08$
1000	$79.72\pm 0.80$	$82.60\pm 1.77$	$81.32\pm 1.56$	$80.51\pm 0.88$	$81.20\pm 0.97$	$84.32\pm 1.68$

**Table 16:** Performance of the random vector functional link with the varied number of enhancement nodes based on deep feature $Fea_{24+64}$

Number of	Sensitivity	Specificity	Precision	$F1_{score}$	Accuracy	AUC
hidden						
neurons						
40	$79.67\pm 2.37$	**83.12±3.50**	**81.87±2.72**	80.70±0.79	**81.44±1.01**	$88.98\pm 0.72$
80	$79.89\pm 2.05$	$82.81\pm 3.63$	$81.64\pm 2.89$	${\textbf {80.70}}\pm {\textbf {0.64}}$	$81.39\pm 1.02$	$89.03\pm 0.90$
100	**80.11±2.35**	$82.39\pm 3.84$	$81.33\pm 2.96$	$80.65\pm 0.74$	$81.28\pm 1.08$	$89.00\pm 0.81$
200	$79.72\pm 1.61$	$82.91\pm 3.19$	$81.66\pm 2.63$	$80.65\pm 1.11$	$81.36\pm 1.34$	$89.03\pm 0.89$
400	$79.67\pm 1.73$	$82.55\pm 2.93$	$81.32\pm 2.33$	$80.45\pm 0.77$	$81.15\pm 0.99$	**89.07±0.91**
800	$79.34\pm 1.80$	$82.91\pm 2.62$	$81.56\pm 2.16$	$80.41\pm 1.00$	$81.17\pm 1.09$	$89.04\pm 0.90$
1000	$79.50\pm 1.91$	$82.86\pm 2.77$	$81.55\pm 2.30$	$80.48\pm 1.08$	$81.23\pm 1.18$	$89.00\pm 0.81$

Similarly, we then varied the number of enhancement nodes to determine the best RVFLN model. The results can be seen in Table 16. As can be seen, RVFLN with 40 hidden neurons is the best model compared to other RVFLN models. Also, the model with 40 hidden neurons showed the highest specificity, precision, $F1_{score}$, and accuracy that justify the effectiveness of the model. However, compared to the RVFLN models that were trained on $Fea_{24}$ and $Fea_{64}$, the RVFLN models trained on $Fea_{24+64}$ are more quite similar, and therefore the benefit of concatenating features from different levels is minimal here.

**Fig. 7:**
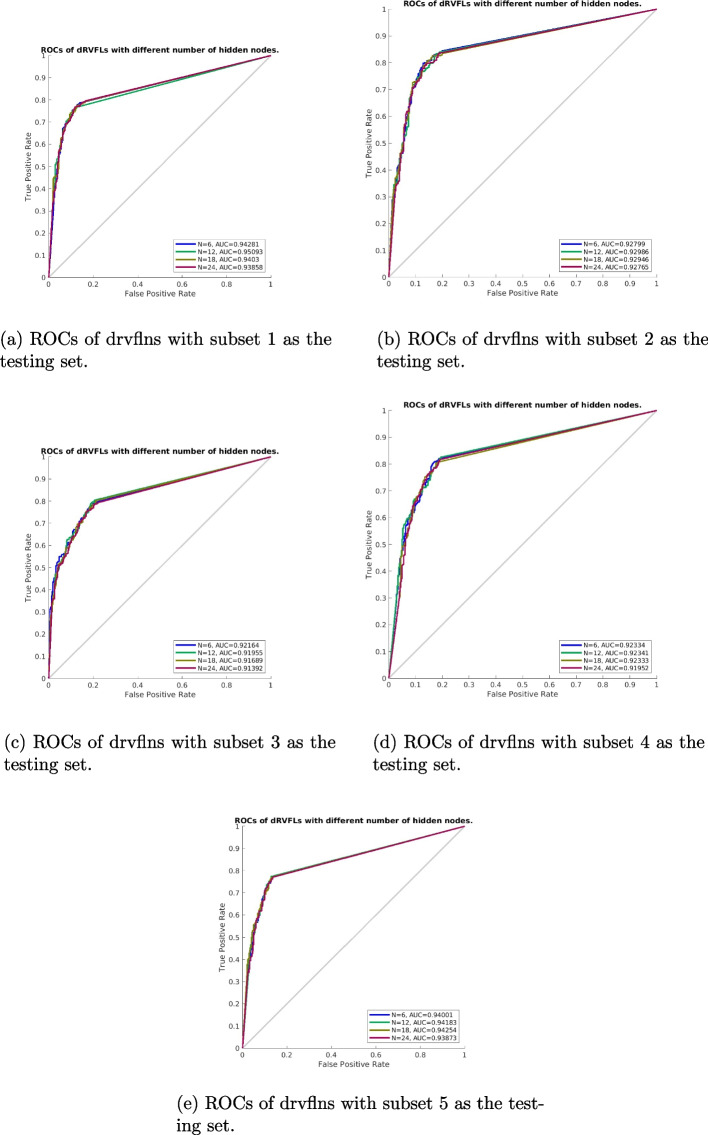
ROCs of drvflns trained with $Fea_{24+64}$ on DDSM

We then varied the number of hidden neurons in each hidden layer while making sure the hidden neurons between the hidden layers were the same for dRVFL models. The classification results can be seen in Table , while the corresponding ROCs can be seen in Fig. 7. As can be seen, the dRVFL model with 12 hidden neurons was the best model compared to other dRVFL models. Specifically, the overall accuracy has been improved to $81.71\%$ with improved sensitivity of $83.17\%$. When these trained models are compared with the models trained on $Fea_{24}$ and $Fea_{64}$, we found that no significant improvement can be seen, while some models showed even worse performance. Nevertheless, the dRVFL model with 12 hidden neurons can be taken as the classifier for breast mass classification.

**Table 17:** Performance of the deep random vector functional link with the varied number of enhancement nodes based on deep feature $Fea_{24+64}$

Number of	Sensitivity	Specificity	Precision	$F1_{score}$	Accuracy	AUC
enhancement						
neurons						
6	$82.49\pm 3.29$	**80.39±2.88**	**81.66±1.84**	$82.03\pm 1.34$	$81.47\pm 1.15$	$93.12\pm 0.97$
12	**83.17±4.06**	$80.17\pm 3.23$	$81.64\pm 1.84$	**82.33±1.42**	**81.71±1.06**	**93.31±1.31**
18	$82.28\pm 3.03$	$80.11\pm 2.30$	$81.39\pm 1.36$	$81.80\pm 1.21$	$81.23\pm 0.96$	$93.05\pm 1.09$
24	$81.71\pm 3.15$	$80.28\pm 2.54$	$81.41\pm 1.69$	$81.52\pm 1.53$	$81.01\pm 1.36$	$92.77\pm 1.11$

For SNN models, we then varied the number of hidden neurons. The corresponding results can be seen in Table 18. As can be seen, the SNN model with 40 hidden neurons is the best model compared to other SNNs with different numbers of hidden nodes. Strangely, the performance of the SNN model with 1000 hidden neurons experienced the greatest decline. The reason could be the overfitting problem because of large volumes of the parameters introduced by 1000 neurons. However, The comparison between SNN models trained on $Fea_{24}$ and on $Fea_{64}$ showed that the models trained on $Fea_{64}$ have the best performance. Nevertheless, the SNN models are likely to provide results with high sensitivity. So far, all experiments showed that the models trained on $Fea_{64}$ are more likely to perform better than the models trained on $Fea_{64}$ and $Fea_{24+64}$, although the model with the best performance was obtained after being trained on $Fea_{24+64}$. Moreover, dRVFL models showed overall higher performance compared to other classifiers throughout the different settings. Therefore, we believe that the combination between VGG19 based feature extractor and the dRVFL model-based classifier seems to be more reasonable.

**Table 18:** Performance of the spiking neural network with the varied number of hidden nodes based on deep feature $Fea_{24+64}$

Number of	Sensitivity	Specificity	Precision	$F1_{score}$	Accuracy
hidden					
neurons					
40	$79.17\pm 3.77$	$82.86\pm 5.27$	$81.71\pm 3.86$	**80.28±0.68**	**81.07±1.08**
80	$78.34\pm 3.46$	$82.91\pm 5.15$	$81.58\pm 3.91$	$79.80\pm 0.99$	$80.69\pm 1.36$
100	$79.17\pm 4.78$	$81.71\pm 6.36$	$80.83\pm 4.52$	$79.78\pm 0.66$	$80.47\pm 1.12$
200	$79.50\pm 4.04$	$82.13\pm 5.75$	$81.18\pm 4.14$	$80.17\pm 0.76$	$80.85\pm 1.24$
400	$77.96\pm 4.93$	$82.86\pm 6.74$	$81.66\pm 4.79$	$79.53\pm 0.59$	$80.47\pm 1.18$
800	68.84±10.64	**88.37±3.49**	**85.22±2.57**	$75.62\pm 6.47$	$78.86\pm 3.48$
1000	**84.97±13.78**	51.04±46.39	69.01±18.56	$73.34\pm 7.17$	$67.56\pm 17.13$

**Table 19:** Performance of the traditional classifiers for breast mass classification based on $Fea_{24}$

Model names	Sensitivity	Specificity	Precision	$F1_{score}$	Accuracy
SVM	**79.83±1.46**	$82.55\pm 2.42$	$81.32\pm 2.01$	$80.55\pm 0.99$	$81.23\pm 1.10$
$KNN_{5}$	$79.61\pm 1.64$	$82.76\pm 2.53$	$81.46\pm 2.18$	$80.51\pm 1.23$	$81.23\pm 1.32$
$KNN_{10}$	$79.17\pm 2.34$	**83.54±2.33**	**82.07±2.07**	**80.57±1.57**	**81.42±1.49**
$KNN_{20}$	$79.72\pm 2.32$	$82.70\pm 2.33$	$81.43\pm 2.00$	$80.54\pm 1.51$	$81.25\pm 1.44$
DT	$78.23\pm 2.09$	$82.60\pm 1.37$	$81.02\pm 1.02$	$79.59\pm 1.10$	$80.47\pm 0.88$
$BDT_{5}$	$79.50\pm 1.81$	$83.02\pm 2.35$	$81.66\pm 2.07$	$80.55\pm 1.42$	$81.31\pm 1.43$
$BDT_{10}$	$78.95\pm 1.78$	$83.23\pm 1.74$	$81.72\pm 1.62$	$80.30\pm 1.37$	$81.15\pm 1.30$
$BDT_{20}$	$79.39\pm 1.84$	$82.86\pm 2.33$	$81.51\pm 1.98$	$80.41\pm 1.22$	$81.17\pm 1.23$

**Table 20:** Performance of the traditional classifiers for breast mass classification based on $Fea_{64}$

Model names	Sensitivity	Specificity	Precision	$F1_{score}$	Accuracy
SVM	$80.00\pm 1.39$	$82.49\pm 2.34$	$81.30\pm 1.98$	$80.63\pm 1.15$	$81.28\pm 1.24$
$KNN_{5}$	**80.22±1.92**	$82.65\pm 2.26$	$81.49\pm 1.79$	$80.82\pm 0.84$	$81.47\pm 0.84$
$KNN_{10}$	$79.89\pm 2.12$	$83.38\pm 2.25$	$82.07\pm 1.72$	**80.94±0.73**	**81.68±0.66**
$KNN_{20}$	$79.78\pm 2.02$	$82.91\pm 2.56$	$81.65\pm 2.11$	$80.67\pm 0.99$	$81.39\pm 1.02$
DT	$78.17\pm 1.62$	$82.02\pm 2.06$	$80.51\pm 1.98$	$79.32\pm 1.60$	$80.15\pm 1.58$
$BDT_{5}$	$79.50\pm 1.56$	$81.81\pm 1.87$	$80.60\pm 1.54$	$80.03\pm 1.06$	$80.69\pm 1.05$
$BDT_{10}$	$78.73\pm 1.67$	**83.70±1.75**	82.11±1.59	$80.37\pm 1.22$	$81.28\pm 1.17$
$BDT_{20}$	79.23± 1.68	$83.70\pm 1.50$	**82.19±1.38**	$80.67\pm 1.20$	$81.52\pm 1.12$

### Method comparison

Based on the previous experiments, we validated the effectiveness of the classifier on the breast mass classification task. We first compare the performance of our model with the models based on traditional classifiers. Specifically, we examined the performance of traditional representative classifiers, including SVM, KNN, and DT. The results have been listed in Tables 19, 20, and 21. In these tables, $KNN_{5}$, $KNN_{10}$, and $KNN_{20}$ stand for the KNN with 5, 10 and 20 as *k*, respectively. For BDT, $BDT_{5}$, $BDT_{10}$, and $BDT_{20}$ are the BDT of 5, 10, and 20 bags, respectively. As can be seen from Table 19, almost all of the models except $KNN_{10}$ showed declining performance, within which $KNN_{10}$ showed the overall best performance while SVM gave the highest sensitivity. For general KNN models, a larger *k* doesn’t mean better performance, but it should be carefully chosen, which is supported by the results here. Similar conclusions for the choice of the number of DT bags can be found. The comparison between these models showed that KNN models are preferable to DT and BDTs in terms of overall accuracy, while SVM is the model between them. The experiment results of BDT justify its effectiveness as the ensembles of DT. Compared to the novel classifiers trained on the $Fea_{24}$, the performance of the traditional models dropped with a bigger difference against the performance of trained VGG19 models.

For the traditional classifiers trained on $Fea_{64}$, the overall performance of these classifiers is higher than that of the classifiers trained on $Fea_{24}$ while the average accuracy of the best model achieved $81.68\%$. Therefore, we believe $Fea_{64}$ is preferable to $Fea_{24}$. However, SVM so far doesn’t seem to be a suitable classifier for the classification task here as SVM models still showed declining performance on $Fea_{64}$. For KNN models, it seems that the optimal *k* that can lead to the KNN model with even higher performance is close to 10 but remains to be explored as KNN with 10 neighbors. For DT and BDT, the performance of BDT has improved while the performance of DT remained low. It is worth notifying that BDT of 10 bags showed competitive performance though further exploration is needed. For the models trained on $Fea_{24+64}$, it seems no extra benefit is introduced except for SVM. Therefore, the conclusion that $Fea_{64}$ is the most representative feature amongst $Fea_{24}$, $Fea_{64}$, and $Fea_{24+64}$ is boosted. Combining the performance of all models trained on different datasets, our selected novel classifiers, especially dRVFL, turned out to be the ideal classifiers.

**Table 21:** Performance of the traditional classifiers for breast mass classification based on $Fea_{24+64}$

Model names	Sensitivity	Specificity	Precision	$F1_{score}$	Accuracy
SVM	$79.94\pm 1.33$	$82.70\pm 2.14$	$81.46\pm 1.90$	**80.68±1.28**	$81.36\pm 1.33$
$KNN_{5}$	80.16±2.52	$82.18\pm 2.27$	$81.07\pm 1.70$	$80.58\pm 1.06$	$81.20\pm 0.90$
$KNN_{10}$	$79.83\pm 1.90$	82.81±2.34	**81.55±1.94**	80.66±1.04	**81.36±1.05**
$KNN_{20}$	$79.94\pm 2.18$	$82.65\pm 2.72$	$81.45\pm 2.23$	$80.66\pm 1.17$	$81.33\pm 1.20$
DT	$79.93\pm 1.07$	$79.73\pm 1.76$	$79.73\pm 1.76$	$79.14\pm 1.31$	$79.83\pm 1.36$
$BDT_{5}$	**80.17±1.49**	$82.28\pm 1.83$	81.13±1.58	$80.63\pm 1.06$	$81.25\pm 1.05$
$BDT_{10}$	$78.78\pm 1.85$	**82.86±1.21**	$81.36\pm 0.86$	$80.04\pm 0.85$	$80.88\pm 0.63$
$BDT_{20}$	$79.06\pm 1.45$	$82.81\pm 1.66$	$81.38\pm 1.39$	$80.19\pm 0.87$	$80.98\pm 0.85$

Considering the fact that the results in the related works are not replicable due to access limitations, we then compared our methods to the methods that are validated on the same dataset DDSM, as it is a relatively large-scale public dataset when compared to other datasets. The comparison results can be seen in Table 22. In this table, the MultiScaled Deep CNNs proposed by Jing et al. [35] got the highest performance with a sensitivity of 0.97, an accuracy of 0.96, and an AUC of 0.96. However, their work only evaluated 150 test images. The same situation also applied to the work done by Daniel et al. [25]. Then if we compare the performance with the nearest test samples, the work proposed by the Ragab et al. [41] uses 676 test samples that are close to our test samples, our sensitivity increased by 7%, the accuracy increased by 3%, and the AUC increased by 5%. If we compare the work with the Sujata et al. [23], our AUC increased by 3%, compared with Rampun et al. [42], our accuracy increased by at most 2%. In conclusion, our number of images for evaluation became the highest one, and considering the overall performance, it is worth noting that our method has competitive accuracy and AUC against other methods. Combining all factors, including the performance of our model and the size of the validation set, we believe our method turns out to be the most desirable method among all listed methods.

**Table 22:** Performance of the state-of-the-art methods on DDSM

Method	Model name	Number of images	Sensitivity	Accuracy	AUC
		for evaluation			
Daniel et al. [25]	GoogLeNet+	182	0.93	0.93	−
	Data augmentation				
Ragab et al. [41]	AlexNet+ SVM	676	0.76	0.79	0.88
Sujata et al. [23]	DenseNet-121+	−	−	−	0.91
	Data augmentation				
Rampun et al. [42]	Ensemble learning	−	−	$>0.80$	−
Jing et al. [35]	MultiScaled Deep CNNs	150	**0.97**	**0.96**	**0.96**
Our best model	DF-dRVFL	**744**	0.83	0.82	0.93

## Discussion

In this study, we proposed a DF-dRVFL, which includes a deep learning model VGG19-DF and a novel classifier dRVFL for breast mass classification. Through the experiments, we found that the proposed model DF-dRVFL can reach state-of-art performance with less computation power and time. In this section, we discuss the limitations and possible future works based on our proposed DF-dRVFL.

Firstly, the backbone of the deep learning model can be further improved by using more complex and deeper model architectures such as Transformer [53], Ensemble [43], and ConvNeXt [27]. It is also applicable by using some unsupervised learning-based feature extractors instead. For example, the feature extractor can implement by SimClR [3], SimSiam [2], and BYOL [9]. These unsupervised learning-based feature extractors do not need cost on the ground-truth labels and save the training cost further.

The second shortcoming is that the performance of the entire DF-dRVFL highly depends on the feature extractor. If the backbone of the feature extractor cannot perform well on the classification task, the performance improved by the classifiers is meaningless, although there still exists improvements compared with the single feature extractor. In contrast, although some single feature extractors can get higher performance, our proposed dRVFL can still further improve the overall performance and the training time. The computational power is less than the original classifier in the feature extractor.

Another one is how to automatically specify the architectures of the classifiers. As was shown in experiment Section 4, we used the exhaustive-like method to determine the number of hidden neurons or enhancement nodes. However, the models that showed the highest performance on the classification can not be guaranteed as the best model in the search domain. Therefore, the best method for defining the architecture of the classifiers remains to be explored in the future.

The last limitation of this model is the performance of the classifiers. Initially, we evaluated the performance of these classifiers with the original architecture. Therefore, there still exists gaps in performance that can be further enhanced. There are related works that can be deployed, such as ensembling different classifiers [6]. There are different algorithms for ensembling: bagging-based ensemble [10], random forest algorithm [33], AdaBoost [7], voting classifier [44], and so on.

Our future work will be based on the limitations mentioned above to further explore powerful feature extractors and classifiers with breast mass classification tasks. Firstly, we would like to try more complex and deeper feature extractors, then try to implement feature extractors in the unsupervised learning manner in order to save the cost of the labelling process. Secondly, we aim to design an algorithm to automatically explore the numbers of hidden neurons in the proposed classifiers. Thirdly, we will use ensemble-based algorithms to integrate different classifiers to achieve better performance than the single classifier.

## Conclusion

Breast mass severity classification plays an important role in mammogram-based cancer analysis. In this paper, we developed a novel framework DF-dRVFL for breast mass in mammography images. The proposed method is a hybrid architecture deploying using both deep learning architecture and novel machine learning models for the classification task. Based on the experimental results on DDSM, the proposed framework showed very high performance on the datasets. As it is a time-consuming task to train the deep learning model so that the best performance can be achieved, we circumvented the non-trivial task by repurposing the trained deep learning models as the deep feature extractor and introducing novel classifiers instead. After the introduction of the novel classifiers, including dRVFL, the overall training time has been greatly reduced, while the classification performance has also been improved. For the classifiers chosen for this task, we aimed to introduce classifiers that have a good generalizability and novelties in the architectures. To fully use the deep features from different levels, we proposed to combine the deep features from different levels of the trained deep learning models as the input for the classifiers. Hence, dRVFL, which contributed to our DF-dRVFL model amongst all evaluated models, is the model that benefits most from the concatenated features. Also, the experiments showed that $Fea_{64}$ might be more suitable for model training as the models trained via $Fea_{64}$ generally showed higher performance compared to those models trained via $Fea_{24}$. In conclusion, we believe the developed DF-dRVFL model could serve as a promising model for breast mass classification.
